# Supplementary material for: Interleukin-36 cytokines alter the intestinal microbiome and can protect against obesity and metabolic dysfunction
Source: Nat Commun. 2019 Sep 5;10:4003. doi: 10.1038/s41467-019-11944-w (PMC6728358; doi:10.1038/s41467-019-11944-w)
Supplement: Supplementary file 1 — Supplementary Information [file 41467_2019_11944_MOESM1_ESM.pdf]

Interleukin 36 cytokines alter the intestinal microbiome and can protect against obesity and metabolic dysfunction.

Giannoudaki et al.

Supplementary Figure 1

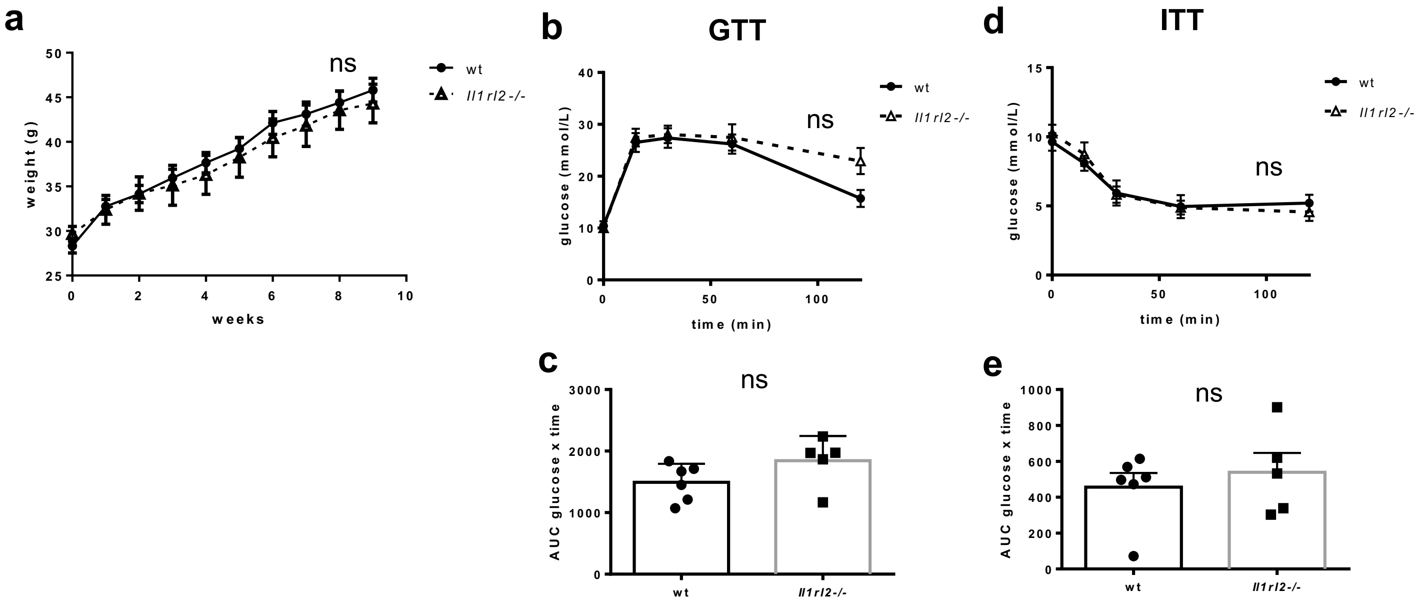

Supplementary Figure 1. *Il1rl2-/-* mice exhibit comparable HFD induced weight gain and glucose/insulin intolerance with wild type controls. a-e Male *Il1rl2-/-* and wt mice of the same age were fed a HFD (60 kcal% fat) for 10 weeks starting from 8 weeks of age (n=5 or 6 per group, representative of 2 independent experiments). **a** Weights measured weekly. **b-c** GTT on wt and *Il1rl2-/-* mice after 8 weeks in HFD, glucose over time (**b**) and AUC (**c**) shown. **d-e** ITT on wt and *Il1rl2-/-* mice after 9 weeks in HFD, glucose over time (**d**) and inverted AUC (**e**) shown. Data represent means ± SEM. Statistical analysis using two-tailed unpaired student's t-test, or RM two-way ANOVA for **a**, **b** and **d**. ns $p > 0.05$

Supplementary Figure 2

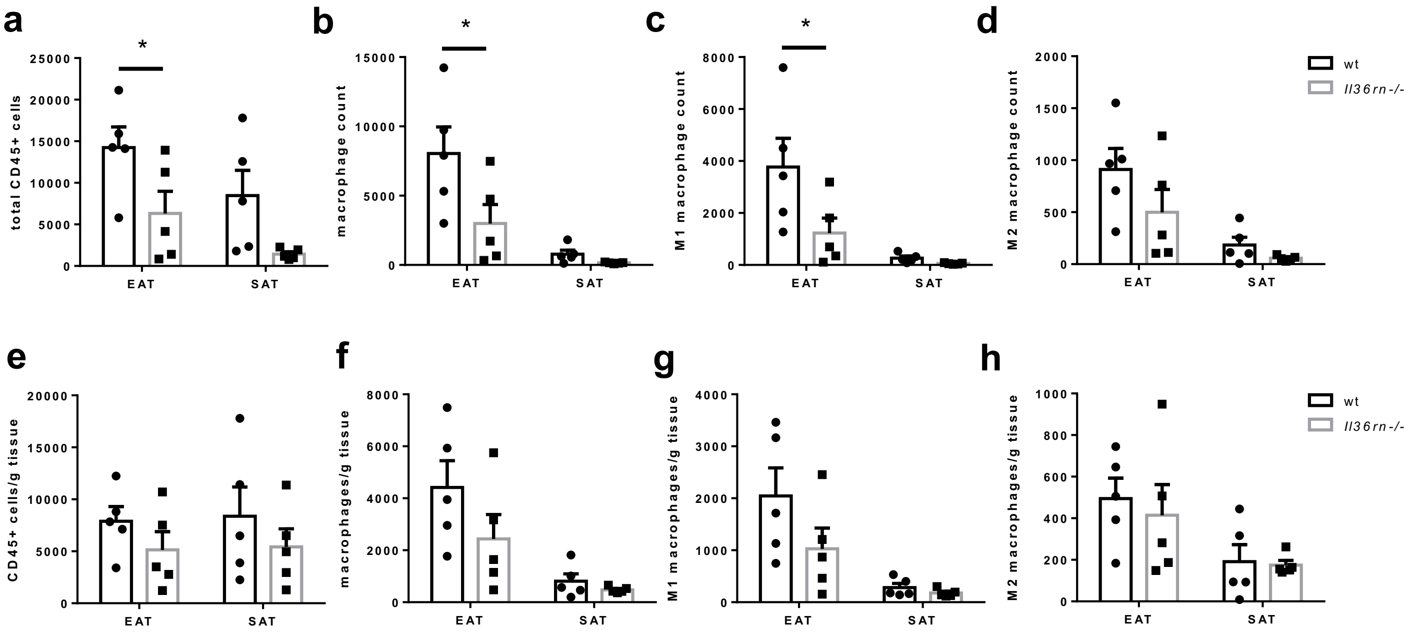

Supplementary Figure 2. Immune cell infiltration in adipose tissue of *Il36rn-/-* and wild type mice after HFD exposure. a-d Absolute numbers of total CD45+ cells (**a**), F4/80+ CD11b+ total macrophages (**b**), F4/80+ CD11b+ CD11c+ CD301- M1 macrophages (**c**), and F4/80+ CD11b+ CD11c- CD301+ M2 macrophages (**d**) in EAT and SAT depots of wt and *Il36rn-/-* mice on HFD for 10 weeks (n=5). **e-h** Normalized cells per g of tissue, for total CD45+ cells (**e**), F4/80+ CD11b+ total macrophages (**f**), F4/80+ CD11b+ CD11c+ CD301- M1 macrophages (**g**), and F4/80+ CD11b+ CD11c- CD301+ M2 macrophages (**h**) in EAT and SAT depots of wt and *Il36rn-/-* mice on HFD for 10 weeks (n=5). Data show means ± SEM. Statistical analysis using two-tailed unpaired student's t-test. * $p < 0.05$

Supplementary Figure 3

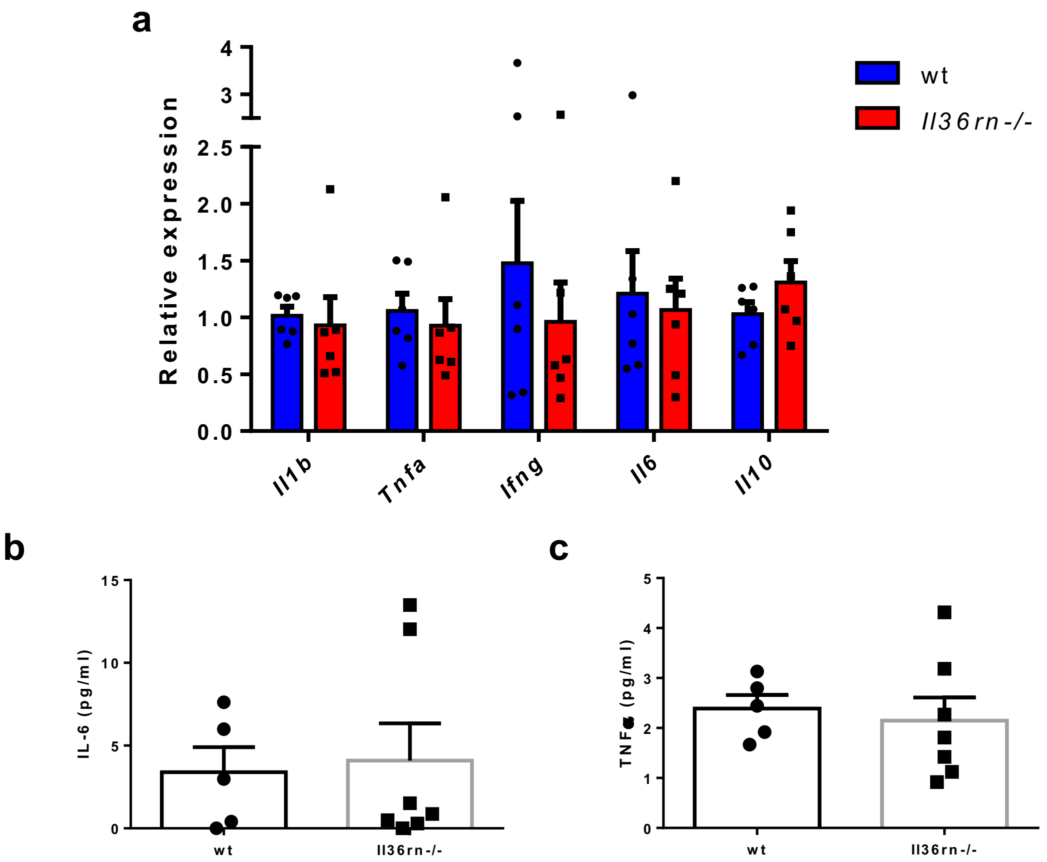

Supplementary Figure 3. Expression levels of inflammatory mediators in the colon and serum of *Il36rn-/-* mice. a Relative gene expression of *Il1b*, *Tnfa*, *Ifng*, *Il6*, and *Il10* in the distal colon of wt and *Il36rn-/-* mice. **b-c** Protein concentration as measured by ELISA of IL-6 and TNFα in the serum of wt and *Il36rn-/-* mice after HFD. Data show means ± SEM. Statistical analysis using two-tailed Mann-Whitney test for **a** and **b** and two-tailed unpaired student's t-test for **c.**

Supplementary Figure 4

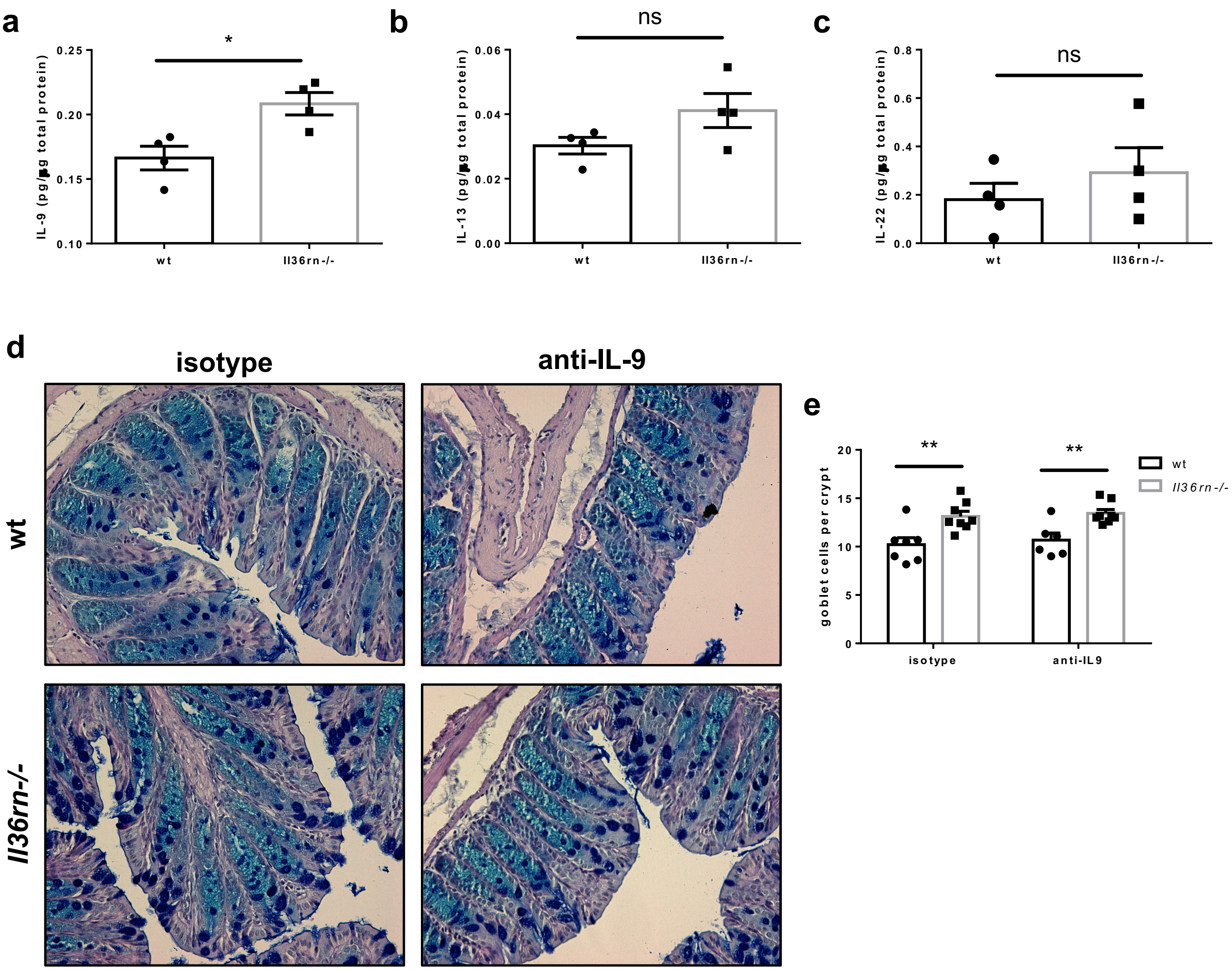

Supplementary Figure 4. Detection of colon cytokine secretion and IL-9 neutralization in *Il36rn-/-* mice. a-c Concentration of secreted IL-9 (**a**), IL-13 (**b**) and IL-22 (**c**) from distal colon explants normalized for total colon tissue protein amount, and measured by ELISA (n=4 per group). **d-e** Representative micrographs of colon sections stained with AB/PAS (**d**) and enumeration of average goblet cells per crypt (**e**) in the colon of wt and *Il36rn-/-* mice after repeated injections of anti-IL9 or isotype control antibody every other day for 5 days (n=6-8 per group). Data show means ± SEM. Statistical analysis using two-tailed unpaired student's t-test**.** ns $p > 0.05$, * $p < 0.05$, ** $p < 0.01$

Supplementary Table 1. Patient Cohort information.

	**Lean (n=37)**	**Obese non-diabetic (n=51)**	**Obese diabetic (n=16)**
**BMI, Kg/m2**	22.8 (18-26.2)	51.8 (37-78) ***	49.2 (33.9-65) ***
**Weight (kg)**	65.8 (48.5-85)	145 (100.6-181) ***	143.4 (94.5-196) ***
**HbA1c, mmol/mol**	33.7 (28-42)	36.8 (29-46) *	66.9 (48-108) ***
**FBG, mmol/liter**	4.9 (3.1-6.3)	5.3 (3.3-7.1)	10.8 (4.7-18) ***
**Cholesterol, mmol/liter**	5.0 (3.4-7.8)	5.1 (3.2-7.2)	4.6 (2.6-6.6)
**Triglycerides, mmol/liter**	1.1 (0.6-2.5)	1.5 (0.6-5.5) *	2.8 (0.7-10.8) ***
**LDL, mmol/liter**	2.8 (1.5-5.5)	3.1 (1.4-5.5)	2.4 (0.8-3.8)
**HDL, mmol/liter**	1.7 (1.1-2.8)	1.3 (0.9-2.2) ***	1.1 (0.8-1.8) ***
**Hypercholesterolemia**	1/37	3/51	10/16
**Hypertension**	4/37	14/51	9/16
**M/F**	14/23	12/39	8/8
**Age**	39.1 (22-65)	43.4 (19-66)	50.6 (36-72.5)
**Medications**	Antibp (4/37)	Antibp (12/51); Others (18/51)	Metformin (9/16); Insulin (3/16); Antibp (9/16)

Clinical data shown as mean with range of values recorded in parentheses. Clinical parameters were compared between Lean and obese non-diabetic, or obese diabetic patients, by Mann-Whitney U test and significant p-values obtained were represented as: * $p<0.05$, ** $p<0.01$, *** $p<0.001$.

Supplementary Table 2. TaqMan Gene Expression Assays used.

GENE NAME	ASSAY ID
Il1f6 (Il36a)	Mm00457645_m1
Il1f8 (Il36b)	Mm01337546_g1
Il1f9 (Il36g)	Mm00463327_m1
Il1rl2 (Il36 receptor)	Mm00519245_m1
Muc2	Mm01276696_m1
Il1b	Mm00434228_m1
Tnf (Tnfa)	Mm00443258_m1
Ifng	Mm01168134_m1
Il6	Mm00446190_m1
Il10	Mm01288386_m1
